# The soybean root membrane lipids and forage quality data in response to field cultivation on agricultural podzols in boreal climates

**DOI:** 10.1016/j.dib.2019.104055

**Published:** 2019-05-22

**Authors:** Muhammad Nadeem, Thu Huong Pham, Ashley Nieuwenhuis, Waqas Ali, Muhammad Zaeem, Waqar Ashiq, Syed Shah Mohioudin Gillani, Charles Manful, Oludoyin Adeseun Adigun, Lakshman Galagedara, Mumtaz Cheema, Raymond Thomas

**Affiliations:** a School of Science and the Environment, Grenfell Campus, Memorial University of Newfoundland, Corner Brook, A2H 5G4, Canada; b Department of Environmental Sciences, COMSATS University of Islamabad, Vehari 61100, Pakistan; c Agriculture Production and Research, Department of Fisheries and Land Resources, Pasadena, Newfoundland, Canada

## Abstract

The objective of this data in brief article is to represent the associated data set regarding our published paper in Plant Science Nadeem et al., 2019. Data set represent soil acid phosphatase activity, association of individual molecular species of four major lipid classes with soybean forage quality indices when cultivated in boreal podzolic soils under cool climatic conditions. Phosphatidylcholine (PC), phosphatidylethanolamine (PE), phosphatidic acid (PA) and acylated glucosyl betasitosterol ester (AGIcSiE) molecular species grouped the soybean forage quality indices in different quadrants on principal component analyses. Furthermore, the total lipid profile and correlation of major lipid species with forage quality indices are included in this data in brief article. This data set support the main findings described in Nadeem et al., 2019.

**Table:** Specifications table

Subject area	Agriculture
More specific subject area	Agricultural biochemistry
Type of data	Figures and Tables
How data was acquired	Data were acquired from field-based studies conducted on acidic podzolic soils on three farms across Newfoundland, Canada. These farms represent boreal podzolic soils in cool climate production systems. Soybean forage and roots were collected at R3 growth stage and root lipids were analyzed using ultra-high-performance liquid chromatography-hydrophilic interaction chromatography-heated electrospray ionization mass spectrometry (UHPLC-HILIC-HESI/MS). Near Infrared Reflectance Spectroscopy technique (Foss NIR System Model 6500 Win ISI II v1.5) was used to measure the soybean forage nutrition quality.
Data format	Statistical data were analyzed with PCA plots, ANOVA and Pearson's correlation.
Experimental factors	Raw data were used to calculate nmole% of lipid classes
Experimental features	Three agricultural farms were selected across Newfoundland, Canada representing a boreal ecosystem or northern climate to conduct the experiment. Specifically, to assess how soybean root membrane lipidome are remodeled when cultivated on agricultural podzols with varying pH in boreal climates to produce forage with similar nutritional quality as that of podzols limed to neutral pH.
Data source location	Data were collected from St. Johns (47°30′4.99″N 52°46′12.34″W), Lethbridge (48°20′21.72″N 53°49′24.80″W) and Pynn's Brook Research Station (49° 4′21.93″N 57°33′36.51″W), Newfoundland, Canada.
Data accessibility	Data are available within this article
Related Research Articles	Nadeem M, Pham TH, Nieuwenhus A, Ali W, Zaeem M, Ashiq W, Gillani SSM, Manful C, Adigun OA, Galagedara L, Cheema M, Thomas R, 2019. Adaptation strategies of forage soybeans cultivated on acidic soils under cool climate to produce high quality forage. Plan Science (*in Press*)

**Table:** 

**Value of the data** • Multidisciplinary data set obtained from three farm locations across Newfoundland containing podzolic soils with varying pH levels • Data set involves biochemistry, dairy science and agriculture which demonstrate the importance of root lipid in producing higher quality forage on podzolic soils in cool climates of boreal ecosystem • Data set depict the importance of membrane lipid remodeling for sustainable crop production on podzolic soils in boreal ecosystem as part of a strategy to improve food security in the Boreal or northern regions • Data set demonstrates similar result trends and add more context to the results observed in the research article by Nadeem et al., [1].

## 1 Data

This data set contain information regarding soybean root lipids, forage quality indices and correlations among these indices when soybean forage are cultivated at 3 farm sites across Newfoundland containing agricultural podzols with varying soil pH. Data set contains five figures and two tables demonstrating the relationship among lipid molecular species and soybean forage quality indices.

**Fig. 1:**
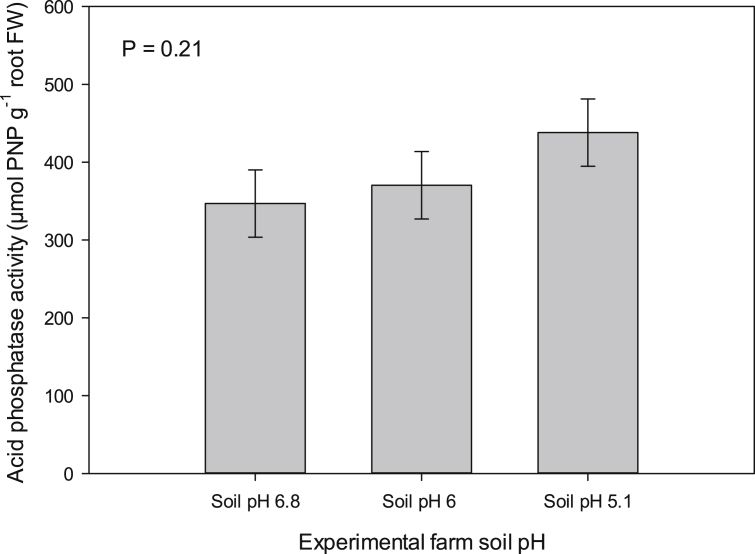
Effect of soil pH on acid phosphatase activity in forage soybean root rhizoshpere when grown under cool climatic conditions.

**Fig. 2:**
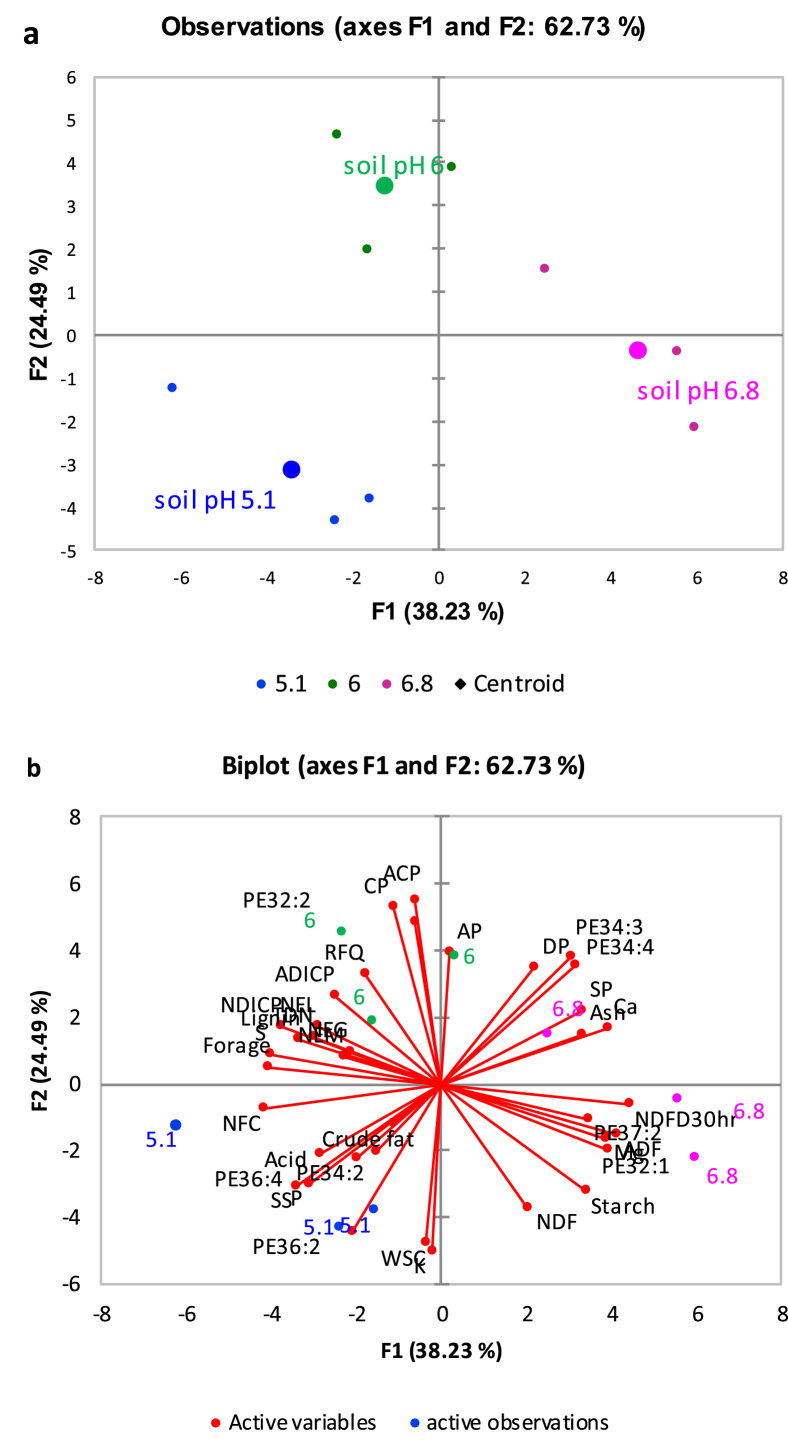
Principal component analysis showing the relationships between root membrane phosphatidylethanolamine (PE) molecular species and forage soybean nutritional quality following cultivation in acidic podzolic soils in cool climate production systems. (a) Observation plot showing segregation of three soil pH based on the centroids on the F1 and F2 axis; and (b) Biplot showing relationship between different observations, forage production, quality and membrane lipid PE at three soil pH.

**Fig. 3:**
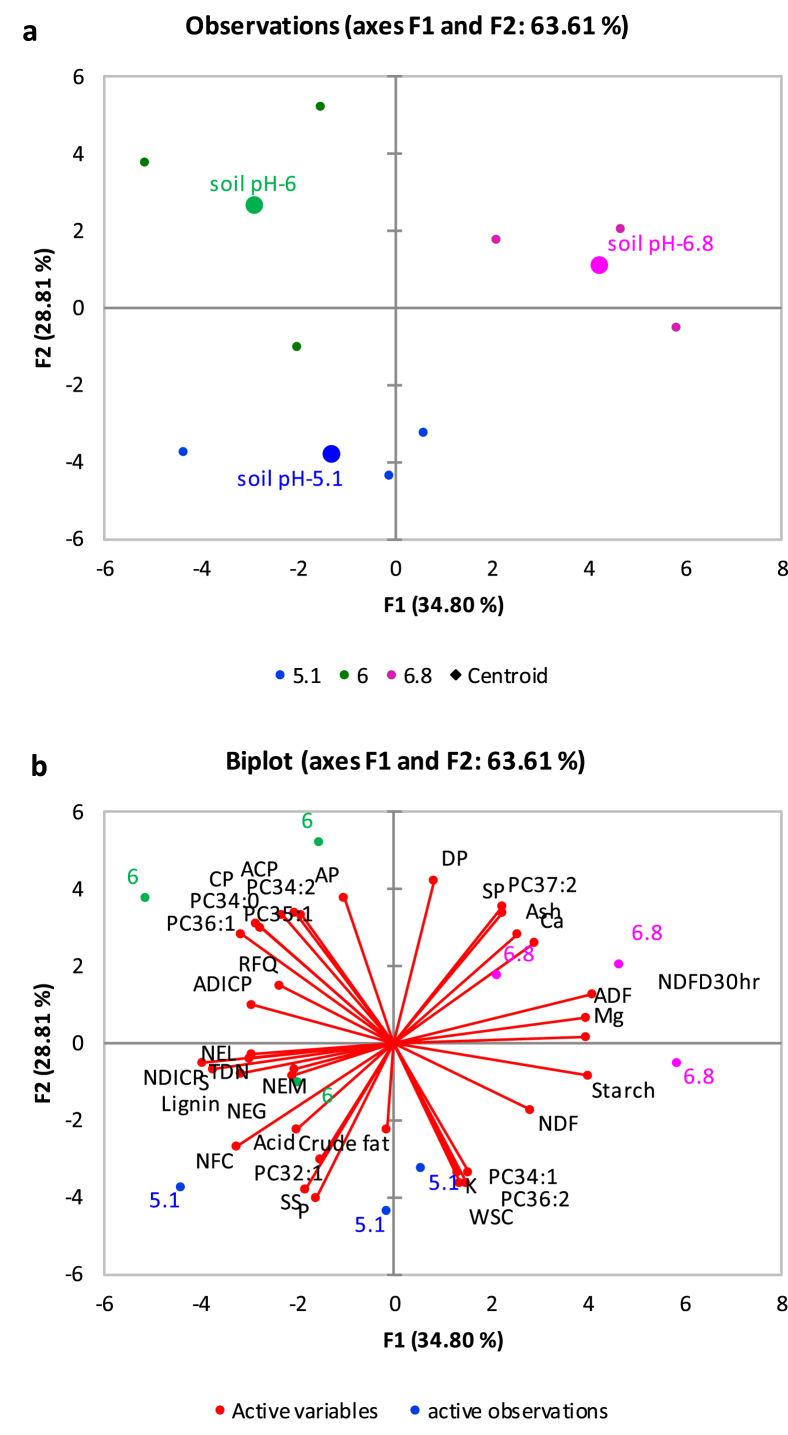
Principal component analysis showing the relationships between root membrane phosphatidylcholine (PC) molecular species and forage soybean nutritional quality following cultivation in acidic podzolic soils in cool climate production systems. (a) Observation plot showing segregation of three soil pH based on the centroids on the F1 and F2 axis; and (b) Biplot showing relationship between different observations, forage production, quality and membrane lipid PC at three soil pH.

**Fig. 4:**
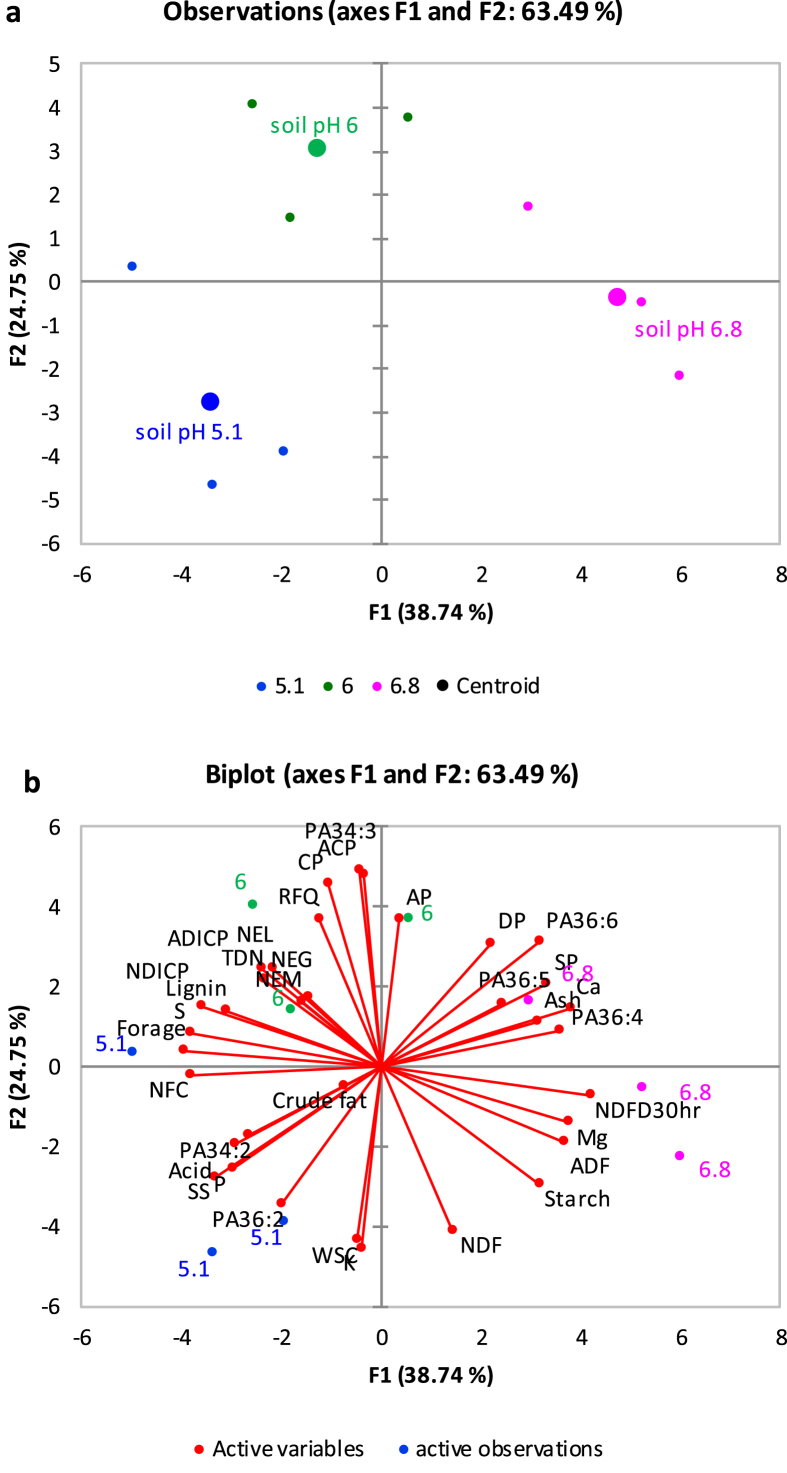
Principal component analysis showing the relationships between root membrane phosphatidic acid (PA) molecular species and forage soybean nutritional quality following cultivation in acidic podzolic soils in cool climate production systems. (a) Observation plot showing segregation of three soil pH based on the centroids on the F1 and F2 axis; and (b) Biplot showing relationship between different observations, forage production, quality and membrane lipid PA at three soil pH.

**Fig. 5:**
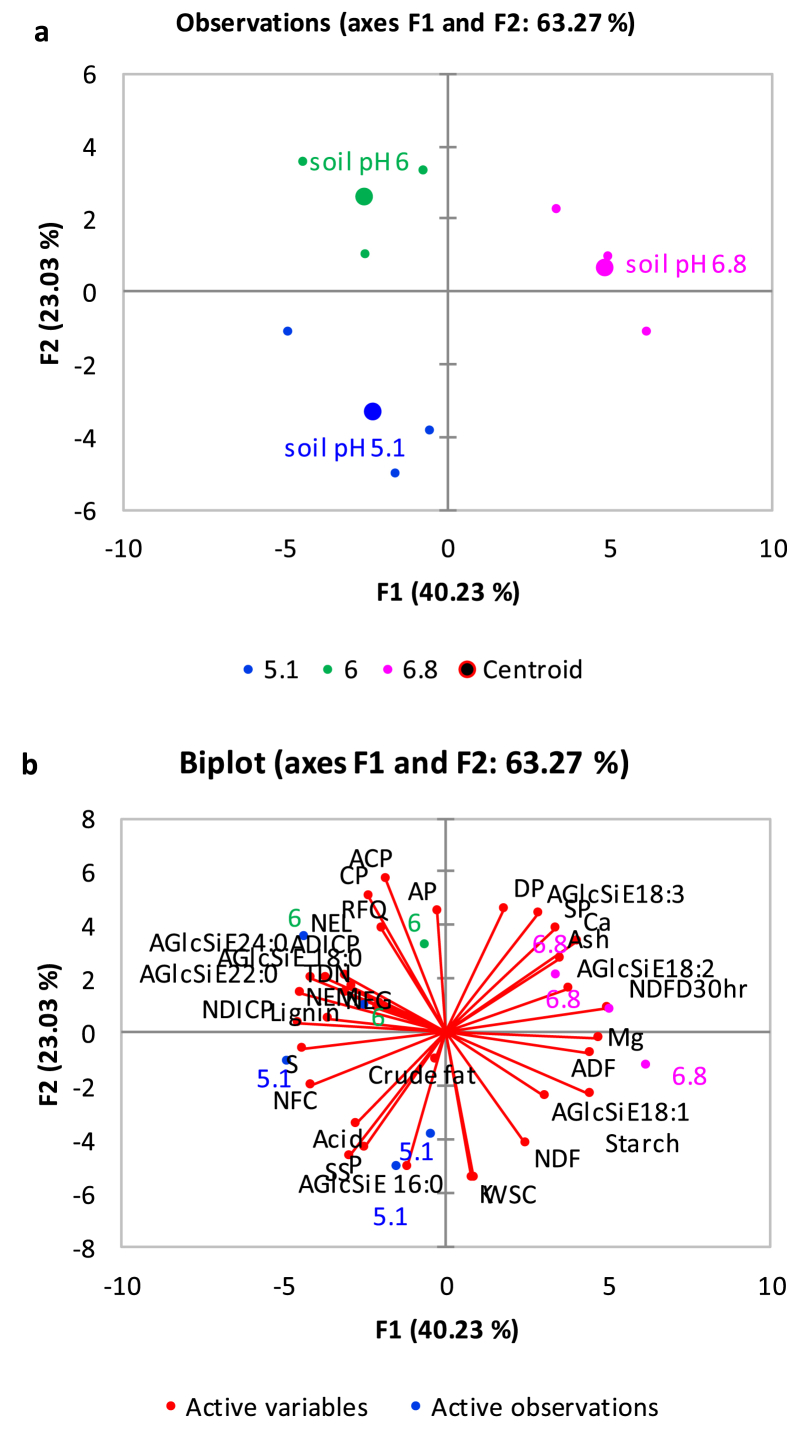
Principal component analysis showing the relationships between root membrane acylated glucosyl betasitosterol ester (AGIcSiE) molecular species and forage soybean nutritional quality following cultivation in acidic podzolic soils in cool climate production systems. (a) Observation plot showing segregation of three soil pH based on the centroids on the F1 and F2 axis; and (b) Biplot showing relationship between different observations, forage production, quality and membrane lipid AGIcSiE at three soil pH.

Figure 1 described the soil acid phosphatase activity in soybean roots rhizosphere across three farm sites. Fig. 2(ab) demonstrates the relationships between root membrane phosphatidylethanolamine (PE) molecular species and forage soybean nutritional quality following cultivation in acidic soil in cool climate production system. The third figure (Fig. 3ab) depicts the relationships between root membrane phosphatidylcholine (PC) molecular species and forage soybean nutritional quality following cultivation in acidic soil in cool climate production system. The fourth figure (Fig. 4ab) shows the relationships between root membrane phosphatidic acid (PA) molecular species and forage soybean nutritional quality following cultivation in acidic soil in cool climate production system. The fifth figure (Fig. 5ab) demonstrates the relationships between root membrane acylated glucosyl betasitosterol ester (AGIcSiE) molecular species and forage soybean nutritional quality following the cultivation in acidic soil in cool climate production system. Table 1 depicts the percent fold changes in root lipid profile compared to neutral pH (6.8) along with three lipid classes. Table 2 shows the correlation among four major lipid species (PE, PA, PC, AGIcSiE) and forage quality indices.

**Table 1:** The effect of soil acidity (pH 6.0 and 5.1) on root membrane lipidome percent fold changes compared to neutral soil pH (6.8) in forage soybeans grown under cool climatic conditions.

Lipid classes	Soil pH 6.0	Soil pH 5.1
PE	19.64	12.56
PA	56.78	16.95
PC	−79.42	−36.17
AGlcSiE	184.59	100.02
PG	−52.58	−3.73
PS	−83.43	21.87
LPC	−44.51	−17.89
SiE	21.97	−29.33
PI	−72.78	−71.55
DGDG	−66.12	−72.08
CL	−81.41	7.64
MGDG	−6.16	−80.91
LPE	−65.53	−21.25
SQDG	82.09	30.19
LPG	−83.16	−61.41
**Phospholipids**	**−11.23**	**−5.42**
**Phytosterols**	**168.36**	**87.11**
**Glycolipids**	**−46.74**	**−73.08**

Values represent the percent fold changes in lipids compared to neutral soil pH (6.8). Lipid class separation was done using hydrophilic interaction chromatography coupled to a high-resolution Orbitrap mass spectrometry. PE = phosphatidylethanolamine, PA = phosphatidic acid, PC = phosphatidylcholine, AGIcSiE = acylated glucosyl sitosterol, PG = phosphatidylglycerol, PS = phosphatidylserine, LPC = lysophosphatidylcholine, SiE = betasitosterol, PI = phosphatidylinositol, DGDG = digalactosyldiacylglycerol, CL = cardiolipin, MGDG = monogalactosyldiacylglycerol, DGDG = digalactosyldiacylglycerol, LPE = lysophosphatidylethanolamine, SQDG = sulfoquinovosyl diacylglycerol, LPG = lysophosphatidylglycerol.

Values of lipid classes were marked as bold.

**Table 2:** Pearson's correlation between four major lipid species and soybean forage production, and forage quality grown on three agricultural farms with different soil pH level under cool climatic production system in Newfoundland, Canada.

Soybean forage quality indicators		PE	PA	PC	AGIcSiE
Lipids	Phosphatidylethanolamine (PE)		**0.62^ns^**	−0.83**	**−0.64^ns^**
	Phosphatidic acid (PA)	**0.62^ns^**		−0.93***	0.83**
	Phosphatidylcholine (PC)	−0.83**	−0.93***		−0.95***
	Acyl sterol glycosides ester (AGIcSiE)	0.75*	0.83**	−0.93***	
Proteins	Crude protein (CP, % DM)	**0.44^ns^**	0.75^*^	−0.69^*^	**0.65 ^ns^**
	Available proteins (% DM)	−0.16 ^ns^	0.26 ^ns^	0.13 ^ns^	−0.18 ^ns^
	Adjustable crude proteins (% DM)	0.21 ^ns^	0.75**	**−0.60 ^ns^**	**0.53 ^ns^**
	Soluble proteins (% CP)	−0.73*	−0.23 ^ns^	0.49 ^ns^	−0.50 ^ns^
	Degradable proteins (% CP)	−0.38 ^ns^	0.13 ^ns^	0.08 ^ns^	−0.07 ^ns^
	NDICP (% DM)	0.74^*^	0.81***	−0.87^***^	0.82***
	ADICP (% DM)	0.18 ^ns^	0.22 ^ns^	−0.30 ^ns^	0.44 ^ns^
Minerals	Potassium (% DM)	−0.19 ^ns^	**−0.56 ^ns^**	**0.50 ^ns^**	**−0.54 ^ns^**
	Calcium (% DM)	**−0.64 ^ns^**	−0.43 ^ns^	0.62 ^ns^	−0.68^*^
	Phosphorus (% DM)	0.28 ^ns^	−0.19 ^ns^	−0.00 ^ns^	0.08 ^ns^
	Magnesium (% DM)	−0.68^*^	−0.74*	0.83**	−0.86***
	Sulfur (% DM)	0.54 ^ns^	**0.57 ^ns^**	−0.69^*^	0.81^***^
Fiber	Acid detergent fiber (% DM)	−0.25 ^ns^	−0.35 ^ns^	0.38 ^ns^	**−0.42 ^ns^**
	Neutral detergent fiber (% DM)	0.16 ^ns^	−0.32 ^ns^	0.16 ^ns^	−0.18 ^ns^
	Lignin (% DM)	0.15 ^ns^	0.16 ^ns^	−0.27 ^ns^	**0.44 ^ns^**
	NDFD 30h (% DM)	**−0.65 ^ns^**	**−0.61 ^ns^**	0.72^*^	−0.74^*^
WS carbs, ash, starch, crude fat	Total digestible nutrient (% DM)	−0.20 ^ns^	0.32 ^ns^	−0.16 ^ns^	0.16 ^ns^
	Starch (% DM)	**−0.55 ^ns^**	−0.84***	0.85***	−0.89***
	Ash (% DM)	**−0.57 ^ns^**	−0.37 ^ns^	0.49 ^ns^	**−0.41 ^ns^**
	Simple sugar (% DM)	0.29 ^ns^	0.01 ^ns^	−0.21 ^ns^	0.37 ^ns^
	Water soluble carbohydrates (% DM)	−0.19 ^ns^	−0.67^*^	**0.46 ^ns^**	−0.25 ^ns^
	Crude fat (% DM)	**−0.53 ^ns^**	**−0.52 ^ns^**	**0.50 ^ns^**	−0.32 ^ns^
	Non-fibrous carbohydrates (% DM)	0.29 ^ns^	0.19 ^ns^	−0.35 ^ns^	**0.48 ^ns^**
Forage production and energy	Forage production (Mg ha^−1^)	**0.61 ^ns^**	**0.50 ^ns^**	−0.69^*^	0.82**
	Net energy for maintenance (Mcal kg^−1^ DM)	−0.17 ^ns^	0.20 ^ns^	−0.06 ^ns^	−0.04 ^ns^
	Net energy for gain (Mcal kg^−1^ DM)	−0.15 ^ns^	0.24 ^ns^	−0.09 ^ns^	0.04 ^ns^
	Net energy for lactation (Mcal kg^−1^ DM)	−0.15 ^ns^	0.29 ^ns^	−0.15 ^ns^	0.15 ^ns^
	Relative forage quality	−0.37 ^ns^	0.11 ^ns^	0.03 ^ns^	0.06 ^ns^

NDICP: Neutral detergent insoluble crude protein.

ADICP: Acid detergent insoluble crude protein.

NDFD: In-vitro NDF digestibility.

Ns: non-significant, * significant at alpha 0.05, ** at 0.01, and *** at 0.001.

Higher correlation values were marked as bold, whereas, bold and italic values were marked significant.

## 2 Experimental design, materials and methods

Experimental design, materials and methods were based on research article *Nadeem* et al.*,* *[1]**, whereas soil phosphatase activity assay is described below.*

### 2.1 Soil phosphatase activity

A half of the soil samples collected from the root rhizosphere was used to determine the soil acid phosphatase activity according to the modified methods of Tabatabai and Bremner [2]. Briefly, a 1 g of 2 mm sieved soil sample was weighed and extracted with 1 mL of 0.09 M citrate buffer with 4.8 pH. Polypropylene centrifuge tubes containing soil and citrate buffer were then centrifuged (Heraeus™ Megafuge™ 16 Centrifuge Series) at 5000×g for 10 min. A 50 μL aliquot of the supernatant was collected and the soil acid phosphatase activity was assessed after incubating for 30 min in an oven (Shel Lab FX14-2, Sheldon Manufacturing Inc. USA) at 37 °C with 1 mM 4-nitrophenyl phosphate and 50 μL citrate buffer. The reaction was terminated immediately after incubation with 20 μL of 0.5 N NaOH. The absorbance was recorded at 405 nm using a spectrophotometer (BioTek Cytation 3 Imaging Reader, USA) and the final enzyme activity presented as μmol pNP g^−1^ of soil per 30 min.
