# Supplementary material for: The soybean root membrane lipids and forage quality data in response to field cultivation on agricultural podzols in boreal climates
Source: Data Brief. 2019 May 22;25:104055. doi: 10.1016/j.dib.2019.104055 (PMC6557726; doi:10.1016/j.dib.2019.104055)
Supplement: Multimedia component 1 [file mmc1.pdf]

Conflicts of Interest Statement

Manuscript title: The soybean root lipids and forage quality data in response to field cultivation on agricultural podzols in boreal climates.

The authors whose names are listed immediately below certify that they have NO affiliations with or involvement in any organization or entity with any financial interest (such as honoraria; educational grants; participation in speakers' bureaus; membership, employment, consultancies, stock ownership, or other equity interest; and expert testimony or patent-licensing arrangements), or non-financial interest (such as personal or professional relationships, affiliations, knowledge or beliefs) in the subject matter or materials discussed in this manuscript.

Author names:

Muhammad Nadeem, Thu Huong Pham, Ashley Nieuwenhuis, Waqas Ali, Muhammad Zaeem, Waqar Ashiq, Syed Shah Mohioudin Gillani, Charles Manful, Oludoyin Adeseun Adigun, Lakshman Galagedara, Mumtaz Cheema,* Raymond Thomas

The authors whose names are listed immediately below report the following details of affiliation or involvement in an organization or entity with a financial or non-financial interest in the subject matter or materials discussed in this manuscript. Please specify the nature of the conflict on a separate sheet of paper if the space below is inadequate.

Author names:

Muhammad Nadeem, Thu Huong Pham, Ashley Nieuwenhuis, Waqas Ali, Muhammad Zaeem, Waqar Ashiq, Syed Shah Mohioudin Gillani, Charles Manful, Oludoyin Adeseun Adigun, Lakshman Galagedara, Mumtaz Cheema*, Raymond Thomas*

This statement is signed by all the authors to indicate agreement that the above information is true and correct (*a photocopy of this form may be used if there are more than 10 authors*):

Author's name (typed)	Author's signature	Date
Muhammad Nadeem	[illegible]	April 16, 2019
Thu Huong Pham	[illegible] Huong Pham	April 16, 2019
Ashley Nieuwenhuis	[illegible]	April 17, 2019
Waqas Ali	Waqas Ali Digitally signed by Waqas Ali Date: 2019.04.17 05:52:32 +05'00'	
Muhammad Zaeem	[illegible]	April 16, 2019
Waqar Ashiq	Waqar Ashiq	April 16, 2019
S. S.M. Gillani	[illegible]	April 16, 2019
Charles Manful	Charles F. MANFUL	April 16, 2019
Oludoyin A. Adigun	[illegible]	April 16, 2019
Lakshman Galagaedara	Lakshman Galagedara [illegible]	
Mumtaz Cheema	Mumtaz Cheema [illegible]	April 15, /2019
Raymond Thomas	[illegible]	
